# Space-qualifying silicon photonic modulators and circuits

**DOI:** 10.1126/sciadv.adi9171

**Published:** 2024-01-05

**Authors:** Dun Mao, Lorry Chang, Hwaseob Lee, Anthony W. Yu, Bennett A. Maruca, Kaleem Ullah, William H. Matthaeus, Michael A. Krainak, Po Dong, Tingyi Gu

**Affiliations:** ^1^ Department of Electrical and Computer Engineering, University of Delaware, Newark, DE 19716, USA.; ^2^ II-VI Incorporated, 48800 Milmont Drive, Milmont, CA 94538, USA.; ^3^ NASA Goddard Space Flight Center, Lasers and Electro-Optics Branch, Greenbelt, MD 20771, USA.; ^4^ Department of Physics and Astronomy, University of Delaware, Newark, DE 19716, USA.

## Abstract

Reducing the form factor while retaining the radiation hardness and performance matrix is the goal of avionics. While a compromise between a transistor’s size and its radiation hardness has reached consensus in microelectronics, the size-performance balance for their optical counterparts has not been quested but eventually will limit the spaceborne photonic instruments’ capacity to weight ratio. Here, we performed space experiments of photonic integrated circuits (PICs), revealing the critical roles of energetic charged particles. The year-long cosmic radiation exposure does not change carrier mobility but reduces free carrier lifetime, resulting in unchanged electro-optic modulation efficiency and well-expanded optoelectronic bandwidth. The diversity and statistics of the tested PIC modulator indicate the minimal requirement of shielding for PIC transmitters with small footprint modulators and complexed routing waveguides toward lightweight space terminals for terabits communications and intersatellite ranging.

## Teaser

Nanoscale optoelectronic devices and circuits may transcend the limits for future space instrumentations.

## INTRODUCTION

Optical communication with terabit capacity is demanded by key applications ranging from near Earth to deep space communications and from human-space exploration to astrophysics experiments (*1*–*3*). Photonic integrated circuits (PICs) with extreme energy efficiency, bandwidth (*4*–*7*), and system-on-chip capacities (*8*–*9*) have emerged as promising candidates for space-borne optical communication instruments and adaptive signal processors (*10*–*14*). Silicon (Si) PICs are foundry manufacturable, and the information exchange and broadcasting are carried out by optical waveguides (WGs), which markedly expands the throughputs compared to copper interconnect and naturally less sensitive to electromagnetic interferences, charge-related total ionizing dose (TID) and transient single-event effects. The displacement damage (DDD) of the particle radiation-induced defects is the primary concern for bulk optoelectronic components. Understanding the PIC’s response to high-energy particle radiation not only facilitates this nanophotonic technology to be infused into future flight missions involving electronic circuits (*15*) and fiber-based technologies (*16*). This knowledge also provides scientific insights toward the explorations of nanoscale photonic modulation of energetic particle beams, particle-photon entanglement, and particle quantum optics (*17*, *18*).

Cosmic radiation covers a wide set of radiation sources with a broad range of energy spectra. Space radiation is composed of two primary groups: cosmic rays of electromagnetic wave packets and radiation particles (protons, electrons, and heavy ions) reaching extremely high kinetic energy (*16*). Ground radiation tests of Si PICs have been focusing on single-frequency γ-ray (10^6^ eV) (*19*–*22*) and x-ray (10^4^ eV) exposure of passive devices (*19*, *23*–*25*), with dosage equivalent to 10^3^ years of exposure on low earth orbit (LEO). The high dosage of such electromagnetic radiation results in primary TIDs in Si-on-insulator (SOI) substrate and reduces the tuning efficiency (*23*, *25*, *26*). Particle radiation (especially protons and heavy ions) is considered the primary cause of degradation for bulk optoelectronics in space (*27*–*29*), but no study has been found on the particle radiation impacts on the nanoscale-doped photonic circuits. Brasch *et al.* (*30*) combine four proton sources for reproducing the particle radiation energy spectra close to LEO and exposed passive SiN*_x_* PICs, but the high-energy portion of the cosmic radiation particles is still missing. We know also that the exposed material is undoped SiN*_x_* PICs rather than the doped Si that we study here. This high-energy proton exposure can lead to cluster defects (*31*) and cannot be shielded even with a 10-mm aluminum (Al) sheet (*30*).

When the high-speed cosmic particles incident on the device’s top surface, their kinetic energy attenuates with their penetration depth. Cascaded atomic structural damage is expected along its trajectory as the energetic particle collides with adjacent and secondary atoms. Monte Carlo simulations capture that these nuclei displacements scale with the particle mass but are inversely correlated with the particle velocity (*32*, *33*). In bulk optoelectronic components, the speed-attenuated orbital protons create nuclei vacancies and displaced atoms in the active layer embedded at least a few micrometers below the top surface, leading to device degradation (*34*, *35*). In optical fibers, increased insertion loss and change in refractive index were observed after cosmic radiation exposure (*36*, *37*). However, the high-energy proton radiation impacts on nanoscale optoelectronic circuits are fundamentally different. Minimal nuclei displacement is expected given orbital protons’ high kinetic energy and limited attenuations when they reach the top device layer of SOI but little is known about the optoelectronic device response to the charged particle interactions with the bounded electrons. In particular, the charged particles’ inelastic scattering with bounded electrons takes place at a much higher kinetic energy (up to 100 MeV) (*38*) compared to nuclei (in kilo- or megaelectron volts).

## RESULTS

**Fig. 1.**
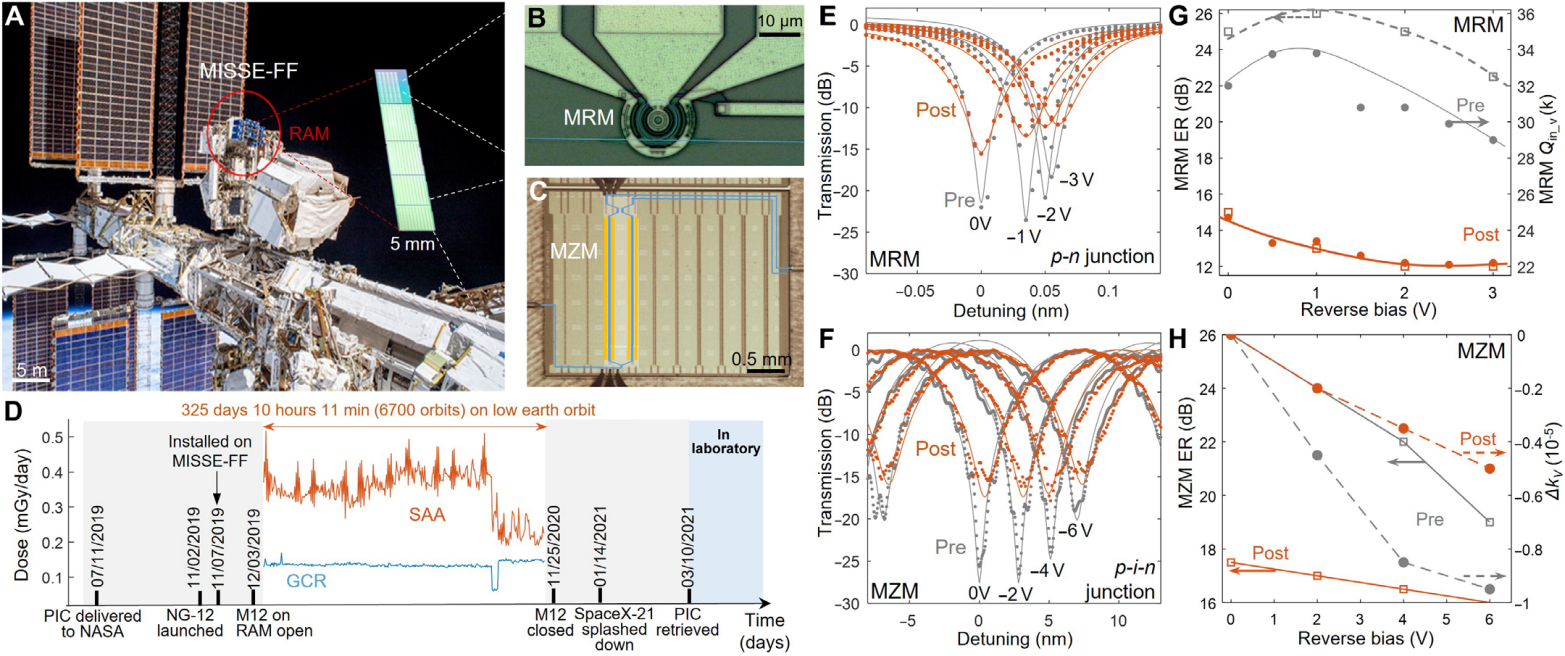
Cosmic radiation exposure on international space station (*7*, *8*). (**A**) Space view of the MISSE-FF material carrier, where the PICs were mounted on the RAM direction. (**B**) Microscope image of the MRM (size of 10 μm) and (**C**) MZM with subcentimeter feature size. Blue lines denote optical WG path. (**D**) Detailed timeline with the daily radiation dosage recorded by a radiation environment monitor installed on ISS. (**E**) Voltage-dependent transmission spectra of the same MRR and (F) MZM pre- and post-flight. Dots are measured data points, and the curves are the theoretical fitting (fig. S1). (**F**) Reverse bias–dependent ER and extracted intrinsic quality factor *Q*_in_ for MRM. (**G**) Reverse bias–dependent ER and extinction coefficient (Δ*k*) for MRM and (**H**) MZM. Dots are obtained by fitting the models to the experimental data. The curves are eye guides. Gray denotes before flight. Orange denotes after flight.

Leveraging the manufacturing capability of Si photonic foundries, we performed a space test of subwavelength photonic devices by exposing arrays of Si PIC devices and circuits on LEO for nearly 11 months. The PICs from different foundries were mounted on the Materials International Space Station Experiment–Flight Facility (MISSE-FF) located near the exterior of the International Space Station (ISS) (Fig. 1A). The radiation dosage is expected to approach the amount received by the optical instruments in CubeSat missions (*3*). Si photonic modulators in micrometer and subcentimeter size (Fig. 1, B and C) were prepared to compare their direct current, continuous wave response, high-speed optoelectronic, and nonlinear optic responses before and after LEO exposure. Limited charge scattering centers are created, given the unchanged carrier mobility, series resistance, and electro-optic tuning efficiency. The substantially reduced free carrier lifetime (τ_rec_), reflected as the expanded optoelectronic bandwidth and reduced thermal nonlinearities, is attributed to the high-energy protons created dangling bonds in the top Si layer on SOI. Through characterizing arrays of doped WGs with different lengths, we observe the evidence of the heavy ion degradation and damaging trajectories along SOI.

We prepared one passive die with arrays of high-*Q* microring resonators (MRRs) from the American Institute of Manufacturing of Integrated Photonics (AIM Photonics), and two active dies with Mach Zehnder modulators (MZMs) and microring modulators (MRMs) from the Institute of Microelectronics (IME) in Singapore for LEO exposure (*39*). Other dies from the same multiproject-wafer (MPW) runs were kept in the laboratory as control samples (Materials and Methods). The samples sent to space are wrapped in paper sleeves and protected under a thin Al sheet (0.110 inch or 2.794 mm) against micrometeorites. Note that the Al shielding effect is very limited for γ-rays and highly energetic particles (>10^7^ eV per particle) that are prevalent on LEO (*30*). Such a thin Al sheet can greatly attenuate most of the low-energy radiation (<1 MeV per particle) in the ground test. The suitcase enclosed those samples unfolded in space, facing toward the direction of the flight (RAM) on ISS. The orbital temperature cycles are stabilized between −30° and +40°C. The exposure lasted 325 days 10 hours and 11 min (approximately 6700 orbits) (Materials and Methods). The daily cosmic radiation dosage was recorded by the Si-based dosimeters installed on ISS, composed of the galactic cosmic radiation (GCR) and south Atlantic anomalous (RAA) (Materials and Methods). The interplanetary particles of GCR are mostly protons (~80%) and α particles (10%), with energies ranging from 10^7^ to 10^11^ eV per particle (*27*). Specifically, the GCR includes heavy ions of more than 50 MeV, created during their path through the interstellar gas. The integral of the daily dosage (GCR and RAA) results in the accumulated total radiation dosage of 148.5 mGy or 14.85 rad (Fig. 1D).

The LEO-exposed MPW dies include arrays of the carrier depletion–based Si photonic modulators, including 10-μm radius MRMs (Fig. 1B) and MZMs with the active arm length of a subcentimeter (Fig. 1C). During the device characterizations, the light was coupled on and off the chip through lensed fiber and edge coupler. The bias and radiofrequency (RF) electronic signals are sent to the chip through high-speed ground-signal-ground (GSG) probes (shown in Fig. 1C) (Materials and Methods). The Si single-mode WGs define the optical paths for making the interferometric circuits (marked as blue lines in Fig. 1, B and C). Different from most of the ground test reports, our experiments show that the electro-optic tuning efficiency remains invariant after radiation exposure for both MRMs (Fig. 1E) and MZMs (Fig. 1F). The models for fitting the transmission spectra of MRMs and MZMs are detailed in the Supplementary Materials section 1.1.. The extinction ratio (ER) drops from near 24 to 14 dB for MRMs (Fig. 1G) and from 26 to 17.5 dB for MZMs (Fig. 1H). By fitting the coupled-mode theory (CMT) model to the transmission spectra (eq. S2), the intrinsic quality factor (*Q*_in_) for the MRM is extracted for pre- and postflight devices (drops from 36,000 to 25,000 at zero bias), indicating a 30% increase in propagation loss α. Reduced sensitivity of ER and extinction coefficient (Δ*k*) to reverse bias is observed in both MZMs and MRMs (Fig. 1H).

**Fig. 2.**
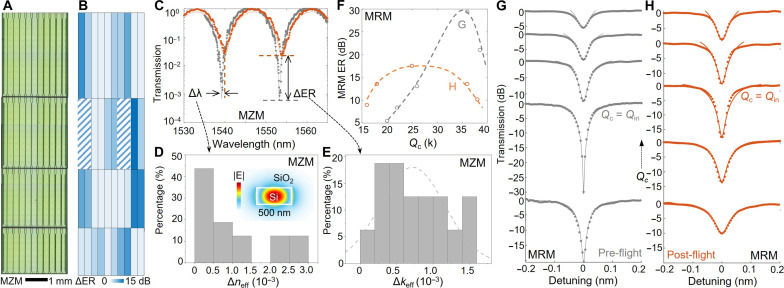
Complex refractive index contrast and modified optical responses in the interferometric Si photonic circuits. (**A**) Optical microscope image of a set of 40 MZMs fabricated on the same die from a multiproject wafer run. (**B**) Corresponding ER reduction (ΔER) after radiation exposure on LEO. (**C**) Representative transmission spectra of the same device on the same die pre- (gray) and post-flight (orange). The measured environmental temperature difference is less than 0.1°. (**D**) Refractive index variation (Δ*n*_eff_) extracted from the resonance drift (Δλ), where the error bar of the temperature variation is around 10^−4^. (**E**) Derived extinction coefficient (Δ*k*) for the same sets of the MZM pre- and post-flight. (**F**) ER versus coupling quality factor (*Q*_c_) of a set of MRMs with the varying gap between the resonator and bus WG. (**G**) Transmission spectra of the MRM before and (**H**) after exposure to LEO.

The propagation loss of active WGs is characterized by measuring the arm length–dependent fiber-to-fiber loss of MZMs (Fig. 2A). The incremental propagation loss induced ER reduction was mapped across an MPW die with more than 40 devices (Fig. 2B). The analytical model provides the device-dependent refractive index (Δ*n*) and Δ*k* change (Fig. 2, C to E). The extracted Δ*k* of 10^−3^ aligns with the directly measured incremental propagation loss of 20 dB/cm (fig. S1A). We also measured passive reference WGs with lengths up to 7 mm. No incremental propagation loss was observed after LEO exposure (fig. S1B). Note that the postflight MZMs in the shaded area in Fig. 2B failed as one of the centimeter-long active arms suffered from an additional loss of more than 10 dB. This WG damage, which is only observed in large-footprint MZMs, aligns with the characteristic behavior of heavy ions (fig. S3). On the same die, arrays of MRRs with varying ring-WG coupling gap sizes and coupling quality factor (*Q*_c_) were included. The ER of MRR is maximized near critical coupling, where the *Q*_in_ is close to *Q*_c_. Measured transmission spectra for a set of MRMs indicate that the critical coupled resonator moved from the one with a larger gap size to the bus WG (higher *Q*_c_) to the one with a smaller gap (Fig. 2F). The *Q*_in_ reduction is verified through CMT fittings of the MRMs arrays (Fig. 2, G and H).

**Fig. 3.**
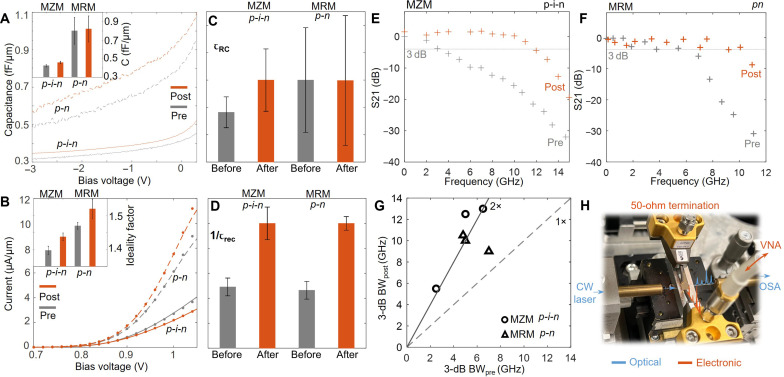
Electronic and high-speed optoelectronic characterizations of Si photonic modulators. (**A**) Exemplary capacitance-voltage curves and (**B**) current-voltage curves for the Si photonic modulators with *p-n* (dashed) and *p-i-n* junctions (solid curves). Insets: Statistical average of normalized capacitance [in (A)] and extracted ideality factors [in (B)] for before exposure (gray) and after exposure (orange). (**C**) Average carrier loss rate by RC constants (τ_RC_) and (**D**) by carrier lifetime (τ_rec_), extracted from (A) and (B), respectively. Error bars represent the SD. (**E**) Measured electro-optic modulation response S21 of an MZM before flight (gray) and after flight (orange). (**F**) S21 comparison for MRM. (**G**) Optoelectronic bandwidth (3 dB) of postflight devices versus preflight devices for the MZMs (circles) and MRMs (triangles). (**H**) Setup for characterizing the high-speed response of MZM.

The impact of the LEO exposure on microelectronic properties is characterized across tens of *p-i-n* junctions in MZM and *p-n* junctions in MRM. The SD of the electronic characterizations among the optoelectronic devices is included as the error bars in Fig. 3 (A to D). No significant change in capacitance or series resistance of the junctions was observed (Fig. 3, A and C), where series resistance is inversely related to carrier mobility (*40*). Increased ideality factor (average value from 1.37 to 1.43) and reduced reverse saturation current (*I*_0_ ∝ τ_rec_^−1/2^) are identified by fitting the IV curves of *p-n* and *p-i-n* junctions (fig. S4) (inset of Fig. 3B) (*40*). The high-speed optoelectronic spectra of that carrier depletion–based Si photonic MZMs and MRMs were carried out using identical experimental apparatus (same power level of optical carrier and RF signal) for pre- and postflight samples (Materials and Methods and Supplementary Materials section S5) (Fig. 3, E to H). Within >30 measurement datasets per device for 12 devices (three pairs of MZMs and three pairs of MRMs), it is conclusive that S21 spectra show well-expanded 3-dB optoelectronic bandwidths across multiple modulators with different device footprints. The faster modulator response, along with the increased ideality factor and reverse saturation current, indicates the reduced τ_rec_. Note that the photon lifetime in MRM is around 5 ps given the *Q*_t_, which is less than 5% compared to the electronic limited response time.

**Fig. 4.**
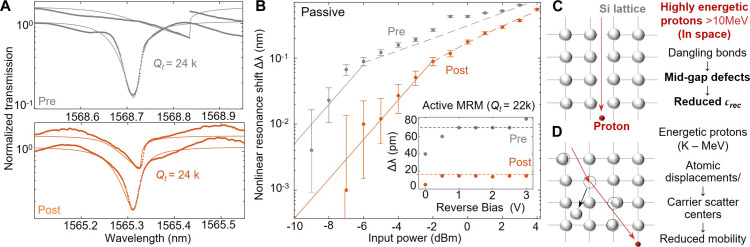
Reduced free-carrier lifetime (τ_rec_) characterized by the photothermal nonlinearities in the MRRs and MRMs. (**A**) Nonlinear transmission spectra for preflight sample (top) and postflight sample (bottom). The two selected MRRs have the same total quality factor (*Q*_t_). Dots: Experimental data. Curves: CMT fits. (**B**) Reduced nonlinear response is characterized by the input power-dependent resonance wavelength detuning to the cold cavity. Gray dots denote pre-flight. Orange dots denote post-flight. Inset: Reverse-biased photothermal nonlinear resonance detuning for pre- and postflight MRR with *p-n* junctions (MRM). Both devices have a *Q*_t_ of 22,000. Preflight samples have higher nonlinear resonance shifts compared to postflight samples. (**C**) Atomistic picture of highly energetic protons (>10 MeV per particle) in space triggered ionization of bounded electrons and thus the mid-gap defects and (**D**) low-energy proton-induced atomic displacement, creating carrier scatter centers and reduce carrier mobility.

The impact of τ_rec_ reduction is also verified through steady-state nonlinear optic measurement in passive MRRs (from AIM Photonics) and active MRMs (from IME). Nonlinear coupled mode equations derive that the nonlinear absorption generated free carrier density linearly increases with the τ_rec_ at steady-state excitations and thus the photothermal effect induced resonance shift (fig. S6) (*41*, *42*). Note that this approximation does not hold at higher optical excitation power well beyond the nonlinear threshold. Comparing the preflight and postflight MRR with the same *Q*_t_ (~24,000), a more significant resonance shift is observed in the preflight sample at the same power levels (Fig. 4A). The power-dependent photothermal resonance shift shows consistent results (Fig. 4B). The reduced τ_rec_ and nonlinear photothermal response are also verified in the MRMs (Fig. 4, inset), where two MRR with similar *Q*_t_ (~22,000) and same *p-n* junction designs were selected for comparison. Because the TID effects are usually recovered after thermal activation, we performed the postflight annealing and examined the τ_rec_ by tracking the nonlinear resonance shift. The nonlinear optical response does not change after heating up to 300°C for an hour (fig. S5). The reduced τ_rec_ is attributed to the dangling bonds formed by the high-energy proton swift through the 220-nm-thick Si layer on SOI (Fig. 4C) (*38*), while the proton with low kinetic energy (slowed down cosmic radiation in bulk device) leads to more extensive damage of nuclei displacement (or DDD) (Fig. 4D). Those displaced nuclei and vacancies act as carrier scattering centers and reduce the carrier mobility. Given the unchanged series resistance and electro-optic tuning efficiency, the carrier mobility remains the same after LEO exposure.

## DISCUSSION

Despite numerous proposals of system-on-chip integration of optical modules for avionics, limited literature is found on space radiation impacts on nanoscale devices, which are critical in propelling nanotechnology for space instrumentation. In addition to the unknown radiation spectra, the device-to-device variations are another challenge blurring the underlying physical mechanism. In this work, we advance the understanding of these issues by sending foundry-manufactured dies with hundreds of passive and active devices beyond 2000-km altitude. Those nanoscale optoelectronic devices and circuits are characterized by the electronic, optical, high-speed optoelectronic, and nonlinear optics aspects. Studies in bulk semiconductor components indicate the charged energetic particles recombine or migrate and form stable defects in the Si lattice structure, resulting in reduced carrier mobility and optoelectronic efficiency (*43*, *44*). No study has been found on the effects of particle radiation on doped PICs. The reduced τ_rec_ in the Si device layer leads to optoelectronic bandwidth improvement of MZMs and MRMs. In contrast, the subcentimer-long active long arm with *p-n* junctions shows a few-decibel excess loss, which results in reduced ER in both MZMs and MRMs. Nevertheless, after long-term exposure, the ER stays beyond 10 dB, providing sufficient modulator amplitude. The tuning efficiency of both types of modulators remains unchanged, unlike many ground radiation exposure results on PIC. The decreased electro-optic tuning efficiency reported in the ground test is induced by the high dosage x-ray or γ-ray exposure (typically megarad), whose mechanism is completely different from the space radiation. In addition, we observed that four adjacent long WGs with *p-i-n* junctions suffered from very high insertion loss after LEO exposure, likely attributed to the accidental deposition of heavy ions and formation of nanoscale cluster defects. The behavior of heavy ions (mostly coming from galactic cosmic rays) aligns with the characteristic of low possibility but disruptive damage to the Si nanowire, including low flux ~4 particles cm^−2^ s^−1^, high atomic number, and high energy. The deposition and reflection of a single heavy ion on Si WG arrays lead to the disconnection of multiple centimeter-long active WGs on the same row.

We summarized the LEO exposure impacts on optical properties (table S1) and optoelectronic response (table S2) of the Si photonic devices, after evaluating nine aspects (listed in table S4) of the nanophotonic device responses over 100 devices. The error bars are included the plots and histograms for refractive index change (Fig. 2, D and E), capacitance (inset of Fig. 3A), ideality factors (inset of Fig. 3B), resistance-capacitance (RC) constant (Fig. 3C), τ_rec_ (Fig. 3D), and doping-dependent propagation loss (fig. S1). Only parameters with pre- and postflight contrast significantly larger than SDs are counted toward LEO exposure impacts. Both MRMs and MZMs exhibit degradation of ER, but electro-optic resonance tuning efficiency remains invariant. The unchanged tuning efficiency and series resistance indicate that the carrier mobility remains the same after LEO exposure. The change of ideality factors, reverse saturation current, and the high-speed optoelectronic response of the embedded *p-n* and *p-i-n* junctions unanimously reflect a nearly half reduction of τ_rec_ (from 500 to 226 ps), aligning with the reduced nonlinear photothermal response in MRRs in both passive and active designs. The τ_rec_ reduction apparently originates from the high kinetic energy protons that form dangling bonds in the Si lattice in SOI. Reduced τ_rec_ increases ideality factors and expands electro-optic bandwidth. While the propagation loss is unchanged in passive WGs, the enhanced propagation loss in the doped Si WGs (+20 dB/cm) might be attributed to the defects formed by the charged radiation (protons and α particles). In addition, accidental disruptive damage on a few MZM with centimeter-long arms is attributed to the cluster defects triggered by the long WG interaction with discrete events of heavy ions. Micro-Raman spectra of the Si transverse optic photon (fig. S2) suggest that the crystal symmetry in passive/undoped Si WGs changed after LEO exposure, while the doped WGs spectra are unchanged.

It is noted that given the timing and expense of the experiments, this experiment focuses on the understanding of nanoscale optoelectronic device statistics with foundry-provided modulators with average performance. The device response time τ_e_ is determined by τ_rec_ and carrier transit time (τ_t_(*V*)): $\frac{1}{\tau_{e}}=\frac{1}{\tau_{\text{rec}}}+\frac{1}{\tau_{\text{tr}}\left(V\right)}$ (detailed in section S5). To clarify the impacts of the τ_rec_, we keep the device operating near-zero bias during the high-speed characterizations. Reverse bias significantly improves τ_tr_ and thus τ_e_, but the impact of τ_rec_ reduction is less visible. It is expected that a higher-speed Si photonic modulator with advanced doping profile engineering is less sensitive to the τ_rec_ reduction (*29*). Consecutive flight experiments can be designed to explore the way diverse radiation particles impact higher-speed Si photonic transceivers.

The small form factor and space radiation hard on-chip active nanophotonic instruments may provide a miniaturized system-on-chip platform for future astrophysics study, earth science observations, and space optical communications. Our research combines the specialties of astronomy and nanoscale optoelectronics, specifying the major impacts on Si photonics from the high-energy orbital particle radiation from the complex space environment. The ground tests with high-density (in kilo- or megarad) x-ray or γ-ray exposure on Si, a-Si, SiC, and SiN*_x_* PICs (table S3) result in totally different types of radiation damage of TID associated with surface oxidation (table S4). In contrast, the LEO cosmic radiation dosage is much lower but carries extremely high-energy particle radiation including protons, α particles, and heavy ions. The lightweight charged particles alter the electronic bonds and mid-gap defects without reducing carrier mobility. Through multimodal characterizations, we conclude that the orbital radiation does not create carrier scattering centers but introduces carrier recombination centers, which result in unchanged driving voltage and reduced bit-error rates for PIC transceivers (table S4), respectively. By avoiding large footprint modulators, those radiation-hard active nanophotonic components can build fully integrated systems with minimal shielding requirements for a year-long orbital operation.

The radiation-tolerant PIC offers a versatile range of applications by supplanting conventional bulk optoelectronic instruments and part of radio frequency transducers. This technology may foster low-cost terabit intersatellite communications, support crewed space explorations, and enable precision astrophysics observations. Moreover, these PICs may find practical utility in LEO CubeSat remote sensing missions of surveying biodiversity, monitoring methane emissions, and assessing natural disasters. Beyond space applications, radiation-hardened PICs can also substitute electronic transducers, particularly for defense and nuclear operations, where highly energetic particle radiation is prevalent.

## MATERIALS AND METHODS

### Foundry-manufactured Si photonic devices

The MRMs and MZMs were manufactured by IME through the MPW run. The lateral *p-i-n*/*p-n* diode configurations were defined by ion implantations: boron for *p*-type (5 × 10^17^ cm^−3^) and phosphorus for *n*-type (5 × 10^17^ cm^−3^). The intrinsic region is lightly *p*-doped (10^16^ cm^−3^). Heavily doped *p*++ and *n*++ regions (1 × 10^20^ cm^−3^) were used to form ohmic contact (*38*, *39*). Vertical vias are patterned and etched on top of cladding oxide for the contact regions, followed by standard Al metallization for direct contact with the heavily doped Si regions.

The photonic structures were defined by a 248-nm-deep ultraviolet photolithography on an 8-inch SOI wafer with a 220-nm device layer, followed by reactive ion etching. Two-step etching leaves a 90-nm-thick Si wing area for supporting the doping and contacts. A thick oxide cover layer is deposited for metal insulation. The passive Si channel WGs are 500-nm wide and 220-nm thick. On each arm of MZM, the thickness of the wing is 90 nm, which supports the doping areas of ridge WGs. Each arm of MZM has an effective modulation WG length that varies among devices. A 42-μm arm-length difference between the arms introduces a free-spectral range of 14 nm. The MRMs have a radius of 5 μm and varying gap distances to bus WGs. Different electronic designs of *p-n* and *p-i-n* junctions were included for each optical design for the MRMs. The MPW runs through AIM Photonics and includes a set of MRRs with radii of 1.5, 2, 3, 5, 10, and 20 μm and varying gaps to bus WGs. The MRRs with a 20-μm radius were selected for comparison with the preflight sample.

### Cosmic radiation dosage counting

The daily radiation dosage is recorded by Si-based radiation environment monitors (REMs) installed on the location COL1A2. Separation of the GCR/SAA dosage follows the Materials and Methods provided for the REMs (*45*). GCR data records all high-energy particles originating from outside of the solar system (interplanetary particles), and SAA records radiation doses from Earth’s inner Van Allen radiation belts closest to Earth’s surface.

### Procedures for exposure on LEO

The samples are assembled in thin paper sleeves and delivered to the Johnson Space Flight Center on 11 July 2019. After assembling all the samples, NG-12 carrying all the payloads (named M12 panel) launched from the Wallops facility (2 November 2019). Nine days later, the NG-12 reached ISS, and the payload was installed on MISSE-FF. On 3 December 2019, the M12 panel was opened toward RAM (direction of the flight) on ISS. M12 was closed on the day of 25 November 2020. SpaceX-21 fetched the M12 from ISS and splashed it down on 14 January 2021. The sample is then retrieved and delivered to the university laboratory on 10 March 2021.

### High-speed optoelectronic measurement

The high-speed response was measured by the optical spectrum analysis method (*46*, *47*). The optical carrier was coupled to the device via edge coupling. RF signal generated from a vector network analyzer (VNA; 0 to 67 GHz) was sent to one port of the MZM transmission line through a high-speed GSG probe (cascade, 0- to 40-GHz bandwidth). The other port of the MZM transmission lines was terminated by another GSG probe, providing a 50-ohm impedance matching. On the same setup configuration, broadband S11 spectra were collected by the VNA.

### Junction characterization

The direct current–voltage traces were obtained by using an Agilent semiconductor parameter analyzer. The voltage-dependent junction capacitance was measured at the same time under 100-MHz small-signal modulation.

### Numerical simulations

The effective index calculation was carried out using Ansys Lumerical MODE solutions.

## Supplementary Materials

### This PDF file includes:

Supplementary Text Figs. S1 to S6 Tables S1 to S4 References (*48*–*60*)
