# Supplementary material for: Space-qualifying silicon photonic modulators and circuits
Source: Sci Adv. 2024 Jan 5;10(1):eadi9171. doi: 10.1126/sciadv.adi9171 (PMC10776012; doi:10.1126/sciadv.adi9171)
Supplement: Supplementary file 1 — Supplementary Text Figs. S1 to S6 Tables S1 to S4 References (48–60) [file sciadv.adi9171_sm.pdf]

Science Advances
AAAS

Supplementary Materials for

Space-qualifying silicon photonic modulators and circuits

Dun Mao *et al.*

Corresponding author: Tingyi Gu, tingyigu@udel.edu; Po Dong, po.dong@ii-vi.com

Sci. Adv. **10**, eadi9171 (2024)
DOI: 10.1126/sciadv.adi9171

This PDF file includes:

Supplementary Text
Figs. S1 to S6
Tables S1 to S4
References

Supplementary file for Space Qualifying Silicon Photonic Modulators and Circuits

S1. Models for the Optical Transmission Spectra

S1.1 Model analysis of Mach-Zehnder Modulator (MZM)

The lineshapes of the transmission spectra of MZM were fitted by the following equations, to extract the complex effective refractive index (n_{eff}):

$$T = \left|\frac{1}{1+\sigma}\left[\sigma \exp\left(j\frac{2\pi}{\lambda_0}n_{eff}(\Delta L + L)\right) + \exp\left(j\frac{2\pi}{\lambda_0}n_{eff}L\right)\right]\right|^2 \qquad \text{(S-1)}$$

σ is the power splitting ratio of the power splitter, which is approximately 1 for the pre-flight device, given the >25 dB extinction ratio (ER) (grey curves in Fig. 1f and Fig. 2c, e). L is the length of the shorter arm, and ΔL = 42μm is the incremental length of the other arm. n_{eff} is the effective index of the single-mode silicon waveguide (WG). After LEO exposure, the effective index changed to $n_{eff\_post} = n_{eff\_pre} + \Delta n + j\Delta k$, where Δn and Δk are the radiation-induced change of real and imaginary parts of the effective index. The propagation loss from the material absorption can be correlated to the extinction coefficient (Δk): $\alpha = 4\pi\Delta k/\lambda_0$. Nearly 10dB reduction of ER (red curves in Fig. 1f and Fig. 2c, e) is attributed to the additional absorption in active WGs.

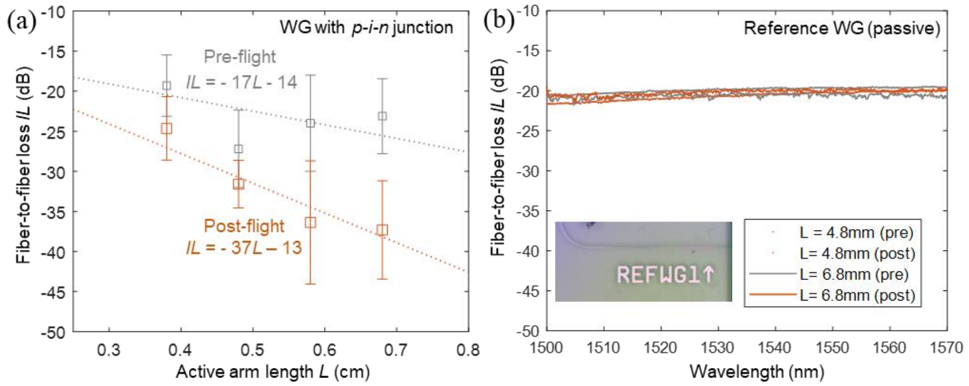

Fig. S1 | Extraction of propagation loss in MZM with varying arm lengths. (a) Fiber-to-fiber insertion loss (*IL*) versus the WG length (*L*) with *p-i-n* junction (>18 devices). (b) Transmission spectra for reference WG defined adjacent to those WG with junctions. Passive WGs do not exhibit noticeable *IL* differences after flight for *L* = 0.48cm and 0.68cm. Bottom inset: optical microscope image of the inverse taper coupler for the reference WG.

This additional absorption is verified by the increased propagation loss (20dB/cm) of the active

arm length (Fig. S1). The fitted propagation loss increases from 17dB/cm (pre-flight, grey) to 37dB/cm (post-flight orange). The total coupling loss (13-14 dB) includes the ones from the power splitters (3dB) and the fiber-chip coupling loss (5dB per facet).

S1.2 Model analysis of Microring Modulator (MRM)

The transmission spectra of the MRM can be fit by the coupled mode theory (CMT) (48):

$$T(\omega) = \left|1 - \frac{1/\tau_c}{-j(\omega-\omega_0)+1/\tau_c+1/\tau_{in}}\right|^2 \qquad \text{(S-2)}$$

where ω_0 is the resonance angular frequency of the MRR and ω is the angular frequency of the input laser. The total cavity lifetime (τ_t) depends on the coupling (τ_c) and intrinsic cavity lifetimes (τ_{in}): $1/\tau_t = 1/\tau_c + 1/\tau_{in}$. $1/\tau_c$ is the loss rates coupled into WG and $1/\tau_{in} = 1/\tau_v + 1/\tau_{lin}$ is the linear loss rate to material absorption ($1/\tau_{lin}$), scattering and radiation ($1/\tau_v$). The relationship between the lifetime and correspondent quality factor is defined as $1/\tau_{c/in/t} = \omega/Q_{c/in/t}$. Given the fixed Q_c for each geometry design, the extinction ratio (transmission T at $\omega_0 = \omega$) depends on Q_{in}: ER (dB) = $20\times[\lg(Q_{in})-\lg(Q_t)]$ (derived from ref. 49), where $1/Q_t = 1/Q_c + 1/Q_{in}$. ER is maximized at $Q_c = Q_{in}$. The propagation loss of the WG can also be easily derived from Q_{in}: $\alpha = 2\pi n_{eff}/(Q_{in}\lambda_0) = \lambda_0/R/FSR$ (50-51). The estimated propagation loss of WG w/ *p-n* junction increases from 19.5 dB/cm to 27.4 dB/cm based on the reduced Q_{in} in MRM. R is the radius of the resonator and FSR is the free spectral range on the MRR transmission spectra. The propagation loss from the material absorption can be correlated to the extinction coefficient (Δk): $\alpha = 4\pi\Delta k/\lambda_0$, which is proportional to $1/\tau_{lin}$.

Table S-I: Impacts on optical properties of silicon photonic devices

Parameters	Dimension	Pre-flight	Post-flight	Difference
Propagation loss of passive Si WG	A few cm long	~3dB/cm	<5dB/cm	Small
Propagation loss of active WGs w/ lateral *p-i-n* junction	A few cm long	17dB/cm	37dB/cm	+20dB/cm
Δn_{eff} extracted from MZM spectra w/ lateral *p-i-n* junction*		--	--	$(1+i)\times10^{-3}$
Q_{in} of passive Si MRR	R = 20μm	130,000	120,000	Small
Q_{in} of active MRM w/ *p-n* junction	R = 10μm	35,000	25,000	Reduced

WG: waveguide; MRR: microring resonator; * Obtained by fitting equation S-1 to the measured spectra (statistics shown in Fig. 2d-e); ** Estimated from the Q_{in} of active MRM.

S2. Micro-Raman Examination

Fig. S2a shows the Stokes component of the reflection micro-Raman spectra of the 500nm wide passive silicon WG, at the excitation laser wavelength of 532 nm. The peak near 521 cm^{-1} is crystalline silicon's main transverse optic (TO) phononic mode. Through fitting the experimental data to the Gaussian model, peak frequency, and full wave half maximum (FWHM) of the measured Raman TO peaks are extracted for passive WGs (Fig. S2a) and active WGs with doping (Fig. S2b). Their statistical responses show the peak position remains nearly invariant after LEO exposure (Fig. S2c). For passive intrinsic silicon photonics devices from AIM, the FWHM expands from 3.7 cm^{-1} (typical for single crystalline silicon) to nearly 4.4 cm^{-1} (Fig. S2e) (52), while the expansion is not observed in the doped (with *p-n* junction) and undoped WGs from IME (Fig. S2f).

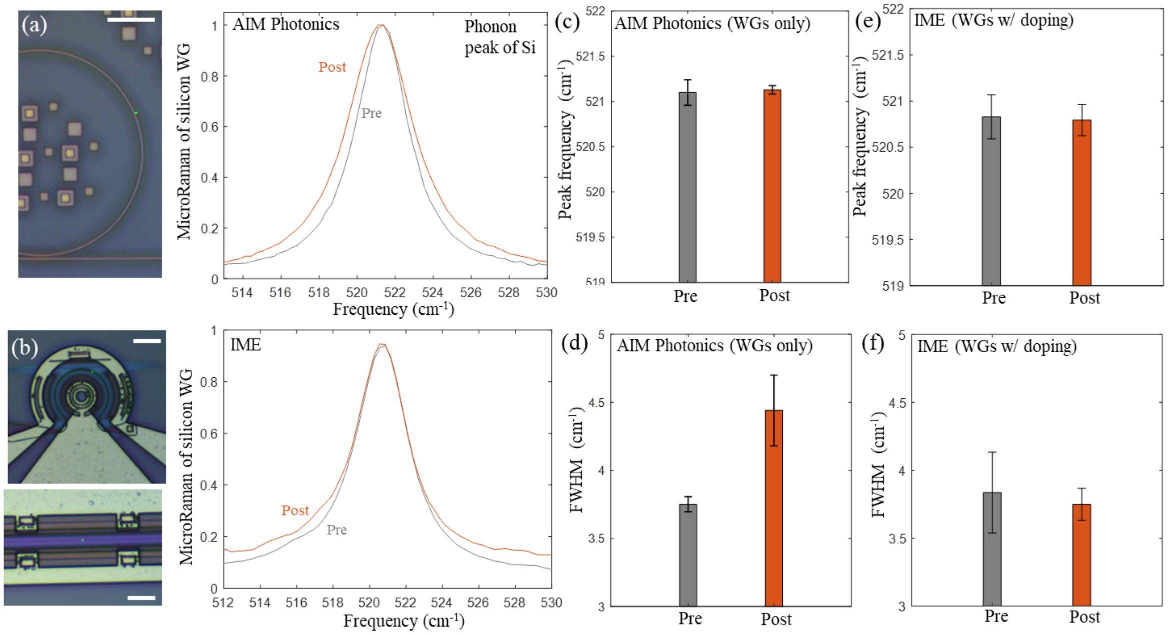

Fig. S2 | Micro-Raman spectra of the silicon WG pre- and post-LEO exposure. (a) Micro-Raman spectra near the optical vibrational modes of the silicon WG from different foundries. Optical microscope image of the probe laser on devices from AIM Photonics Foundry (left inset). (b) Micro-Raman spectra near the WG region for modulators from IME Foundry (left insects). (c) Statistical response of peak frequency for AIM and (d) IME devices. (e) FWHM of AIM and (f) IME devices. Grey: control sample. Orange: post-LEO exposure.

It is noted that the intrinsic region in modulators is smaller (<100nm) compared to the vertically incident probing laser spot size (~500nm), the Micro Raman spectra do not reflect the clean signal from the intrinsic region of active WGs, but primarily of the doped region. The same trend is found for an adjacent reference device close to the MZM without doping. The expansion of the FWHM is associated with the degraded crystal symmetry in single crystalline silicon, which is attributed to particle radiation damage. Such change is only observed in passive WG from AIM photonics. The undoped WG is not affected after LEO exposure. Other weak micro-Raman peaks, such as the second-order peak near 950–100 cm^{-1} remain invariant after LEO exposure.

S3. Impacts of Heavy Ions

Among all the MZMs with long arms, two adjacent pairs of MZMs failed (marked in Fig. 2b). No interference patterns are observed on the transmission spectra, which means one of the active arms is disconnected (Fig. S3). The characteristic of low possibility but disruptive damage to the silicon nanowire aligns with the behavior of heavy ions from GCR, including low flux ~4 particles $cm^{-2}s^{-1}$, high atomic number, and high energy (15). Deposition of a single heavy ion from GCR on a silicon WG leads to a disconnected cm-long active arm in MZM.

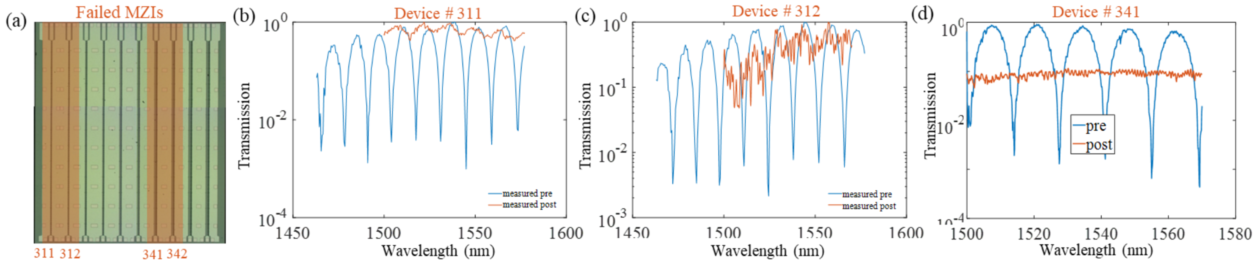

Fig. S3 | Disrupt damage of heavy ions on MZMs with cm-long active arm. (a) Top microscope view of the MPW die with failed MZIs marked on the chip (second row of devices in Fig. 2a). (b) Measured transmission spectra of device 311 before (blue) and after the flight (orange). (c) results for devices 312 and (d) 341.

S4. DC Electronic Response

For both *p-n* and *p-i-n* junctions, the input voltage (V) dependent readout current (I) complies with the empirical diode equation (40):

$$I = I_0\left[\exp\left(\frac{V - I*R_S}{nV_T}\right) - 1\right] + \frac{V}{R_{SH}} \quad \text{(S-3)}$$

Where I_0 is the reverse saturation current, $R_{s/SH}$ is the series/shunt resistance, n is the ideality factor, V_T is the thermal voltage at room temperature (25.9 mV). The reverse saturation current is inversely related to the square root of the minority carrier lifetime (τ_{rec}). The reduced τ_{rec} increases the contribution from the recombination current and thus increases the ideality factor (n). A summary of those parameters impacted by the collective ionizing radiation is listed in Table S1. Reverse saturation current (I_0) and shunt resistance (R_{SH}) are extracted by fitting the reverse biased range of the dark IV curve. The ideality factor n indicates the contributions from diffusion and recombination to the total current (53).

S5. High-speed Optoelectronic Bandwidth for Integrated Photonic Modulators

S5.1. Optoelectronic Bandwidth for MZM (cm length)

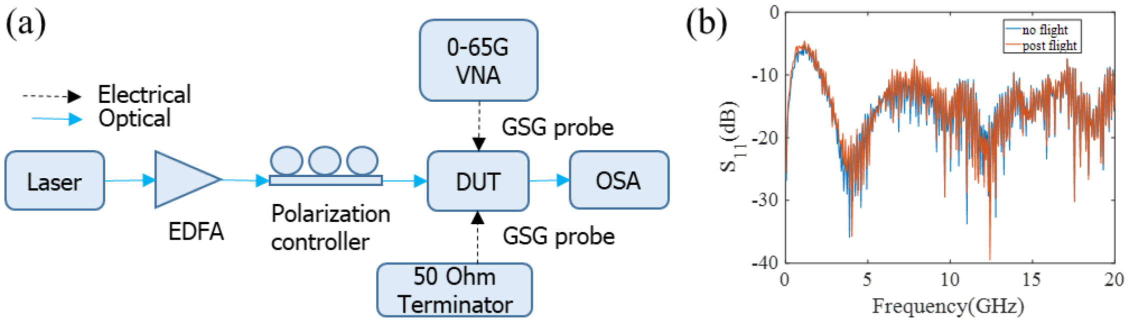

Fig. S4 | High-speed optoelectronic test setup schematics. (a) Details of the optical spectra analysis method for MZM. (b) Simultaneously captured S11 for the RF transmission line of MZM (without optical excitation).

The optoelectronic bandwidth of the MZM is limited by the carrier response time (τ_e) and RC constant (τ_{RC}):

$$\frac{1}{f_{3dB}} = 2\pi(\tau_e + \tau_{RC}) \quad \text{(S-4)}$$

The carrier response time depends on the carrier transient time (τ_{tr}) and τ_{rec} : $\frac{1}{\tau_e} = \frac{1}{\tau_{rec}} + \frac{1}{\tau_{tr}(V)}$, where the carrier transient time τ_{tr} is reverse bias (V) dependent, and the minority carrier recombination lifetime τ_{rec} = 500 ps can be reduced by doping and radiation (in a non-intentionally doped

silicon device layer). The RC constant at low frequency can be estimated by comparing the products of resistance and capacitance (Fig. 3c). The RC constant variation can be estimated by comparing the S11 response (54). The overlapping S11 spectra for pre- and post-flight samples indicate a very limited change in RC constant (Fig. S4).

S5.2 Optoelectronic Bandwidth for MRM (μm size)

The 3dB modulation bandwidth of MRM is limited by the cavity photon lifetime (τ_t), in addition to the electronic design limited response time as MZM:

$$\frac{1}{f_{3dB}^2} = (2\pi\tau_t)^2 + [2\pi(\tau_e + \tau_{RC})]^2 \quad \text{(S-5)}$$

Where τ_t can be derived from the total quality factor of the microring: $\tau_t = Q_t/\omega_0$. Given the extracted total quality factor ($Q_t \sim$ 15k). τ_t is estimated to be around 5 ps, which is much smaller than the electronically limited lifetime.

S6. Free-carrier Lifetime Dependent Nonlinear Photo-thermal Effect in Silicon Microring

At increasing input optical power, the photon excited free carrier and thermal effects impact the light interaction with the MRR. The nonlinear CMT correlating the dynamics of photon, electron, and temperature is (41, 51):

$$\frac{da}{dt} = \left(i(\omega_L - \omega_0 + \Delta\omega) - \frac{1}{2\tau_t}\right) a + \kappa\sqrt{P_{in}} \quad \text{(S-6)}$$

$$\frac{dN}{dt} = \frac{1}{2\hbar\omega_0\tau_{TPA}} \frac{V_{TPA}}{V_{FCA}^2} |a|^4 - N\left(\frac{1}{\tau_{rec}} + \frac{1}{\tau_{tr}(V)}\right) \quad \text{(S-7)}$$

$$\frac{d\Delta T}{dt} = \frac{R_{th}}{\tau_{th}}\left(\frac{1}{\tau_{FCA}} + \frac{1}{\tau_{lin}}\right)|a|^2 + \frac{\Delta T}{\tau_{th}} \quad \text{(S-8)}$$

Where a is the amplitude of resonance mode; N is the free-carrier density; ΔT is the cavity temperature shift. P_{in} is the power carried by an incident continuous-wave laser. $\kappa = \sqrt{\frac{1}{\tau_c}}$ is the coupling coefficient between the WG and cavity, where τ_c is the coupling limited lifetime between the bus WG and MRR. ω_L-ω_0 is the detuning between the laser frequency (ω_L) and cold cavity resonance (ω_0). The total cavity resonance shift is $\Delta\omega = \Delta\omega_N - \Delta\omega_T$, where $\Delta\omega_T$ is the thermal dispersion and $\Delta\omega_N$ is the free-carrier dispersion. The total photon loss rate is $1/\tau_t = 1/\tau_c + 1/\tau_v + 1/\tau_{lin} + 1/\tau_{TPA} + 1/\tau_{FCA}$. The linear loss rate $1/\tau_{lin}$ represents the linear material absorption rate by the mid-gap defect states.

$1/\tau_{FCA}$ is the free-carrier absorption rate. $1/\tau_{TPA}$ is the two-photon absorption rate. V_{FCA} is the effective mode volume for free carriers and V_{TPA} is the effective mode volume for two-photon absorption. In steady-state conditions, the photon amplitude at relatively low input power (P_{in}) can be approximated as $a = 2k\tau_e\sqrt{P_{in}}$. The relationship needs to be rewritten at high input power as $a \propto \tau_e^{-1/5} P_{in}^{1/10}$. Within the low nonlinear intensity region, the input power-dependent carrier density is $N \propto \tau_e P_{in}^2$(derived from equation S-7), and the corresponding incremental temperature is $\Delta T \propto \tau_e P_{in}^3$(derived from equation S-8). We know that the resonance shift ($\Delta\lambda_0$) is thermally induced (ΔT): $\Delta\lambda_0 \propto \Delta T \propto \tau_e P_{in}^3$ is the relation used to calculate the change of the free-carrier lifetime. Given the free-carrier lifetime (500ps) of silicon WG (51), we derived the post-flight free-carrier lifetime of 226.16 ps, by comparing the nonlinear optical resonance shift of MRR with similar quality factor and the same excitation power level. This model is used to interpret the nonlinear transmission spectra in Fig. 4. Note that the total carrier lifetime τ_e is affected by carrier transient time (τ_{tr}) and radiation-dependent recombination lifetime (τ_{rec}) (equation S-4).

Then we proceed to examine the origin of the radiation leading to significantly reduced τ_e. To differentiate between TID and DDD, we bake the sample at increasing temperatures (up to 300ºC) and examine the nonlinear response (Fig. S5). The carrier recombination lifetime seems not recovered after annealing, identifying the source of DDD leading to the reduced τ_e. A higher annealing temperature (typically 600ºC) can heal the dangling bonds (and thus recover τ_{rec}), but also cause permanent damage to doping profiles and electrodes.

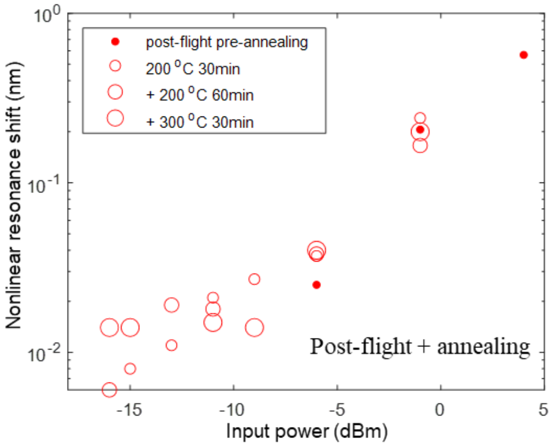

Fig. S5 | Nonlinear resonance shift of the post-flight MRR after accumulative baking.

S7. Summary of the Optoelectronic Response Change after LEO Exposure

The parameters in Tables S-I and S-II are the results from the nanoscale optoelectronic device comparison (same device or representative device in the same MPW run with the same design).

Table S-II: Impacts on the optoelectronic parameters

Parameters	Source	Pre-flight	Post-flight	Difference
Reverse saturation current/leakage current, I_0 (pA)	Dark IV	118	256	++
Series resistance, R_s (Ω)	Dark IV	133*	131*	Trivial
Carrier mobility	$\sim 1/R_s$			Trivial
Shunt resistance, R_{SH} (TΩ)	Dark IV	18.6	16.6	Trivial
Ideality factor	Dark IV	1.39 (*p-n*)<br>1.47 (*p-i-n*)	1.44 (*p-n*)<br>1.51 (*p-i-n*)	Slight increase
Capacitance, C (fF)	C-V	72.2	78.1	Trivial
RC constant, τ_{RC} (ps)	IV & CV	8.46*	7.54*	Trivial
Carrier recombination lifetime (or free carrier lifetime) τ_{rec} (ps)	Nonlinearity in microring	500 (53)	226	<1/2
Optoelectronic bandwidth, f_{3dB} (GHz)	High-speed modulation	~5	~10	×2

* The real values of series resistance and RC constant are likely to be smaller than the estimated values here (extracted from fitting the IV curves upto 1.5V bias).

S8. Comparison to Ground Tests and Defect Type Analysis

Compared to the 18 experiments of the ground radiation tests that we can find (summarized in table S-III), we provided the most comprehensive and systematic studies of those nanoscale optoelectronic devices and circuits, trying to capture the full spectrum of cosmic radiation impacts on nanostructured optoelectronic devices, as well as tracking the atomic origin of the defects, as conventional spectroscopy tools only apply on uniform and low defects single crystalline films. The characterizations are summarized as 9 aspects: (1) utilizing the micro-spectroscopy tool to track the atomic origin of the defects (Section S2); (2) characterized propagation loss through length varying devices (Section S1), (3) effective index variations (Table S-I); (4) revealed doping dependence of radiation impacts by comparing passive device and doped devices with the identical

nanostructures (Section S1 and Table S-I); (5) electro-optic tuning efficiency (Fig. 2, Table S-II); (6) high speed optoelectronic bandwidth (Fig. 3 and Table S-II); (7) detailed electronic characterization and diode analysis by comparing nanostructured *p-n* and *p-i-n* junctions (Fig. 2 and Table S-II); (8) nonlinear optic response of the micro-cavities (Fig. 4) and (9) the influence of the device footprint (from ~10 μm to mm size, Fig. 2).

Table S-III | Comparison of the ground and space radiation test conditions

Ref.	Radiation	Dosage	Energy/ Particle	Material/Device	Radiation impacts	DC EO	RF EO
19	γ	0.1Mrad	10^6 eV	Si MRR	TID ($\Delta n_{eff}\downarrow$)	-	-
20	γ	0.1Mrad	10^6 eV	Si MZI	Propagation loss ↑	-	-
55	γ	0.3 Mrad	10^6 eV	InGaAsP/InP MRR	TID, $\Delta n_{eff}\uparrow$ Q ↓ (-30%) ER ↓	-	-
56	γ	1-15Mrad	10^6 eV	a-Si MRR	TID	-	-
57	γ	10Mrad	10^6 eV	a-Si, SiN_x MRR	TID	-	-
58	γ	10Mrad	10^6 eV	SiC MRR	TID, $\Delta n_{eff}\uparrow$	-	-
21	γ	40Mrad	10^6 eV	Si MRR, MZI	TID, $\Delta n_{eff}\downarrow$	-	Error rate↑
22	γ	100Mrad	10^6 eV	Si MRR	TID	-	-
19	X-ray	6.7Mrad	10^4 eV	Si MRR**	TID, $\Delta n_{eff}\downarrow$	-	-
26	X-ray	10^3Mrad	10^4 eV	Si MRM, MZM (Doped)	TID	Reduced EO tuning	-
23	X-ray	100Mrad	10^4 eV	Si MZM (Doped)	TID, $\Delta n_{eff}\downarrow$	Reduced EO tuning	-
24	X-ray	>10^6Mrad	10^4 eV	Si MZI	TID, $\Delta n_{eff}\downarrow$	--	-
25	X-ray	>10^6Mrad	10^4 eV	Si MRM (Doped)	TID	Reduced EO tuning	-
59	Neutron	2.5Mrad	2 10^7 eV	Si MRM (Doped)	No change	No change	-
23	Neutron	100Mrad	10^7 eV	Si MZM (Doped)	No change	No change	-
59	Neutron	10^{12} /cm^2 (high)	10^7 eV	Si MRR, MZI	No change	N/A	-
30	Proton	100Mrad	4sources	SiN MRR	No change	-	-
60	α	10^{15} cm^{-2}	10^6 eV	SiON MRR	$\Delta n_{eff}\uparrow$, $Q\downarrow$ (-20%)	-	-
This work	LEO (X-, γ-ray, proton, heavy ions)	14.85 rad†	Upto 10^7- 10^{11} eV	Si MRM, MZM (Doped)	Reduced ER (-10dB) and Q (-30%)	No change	Faster
				Si MRR, MZI		Reduced thermal nonlinearity	-

Tilted references: not c-Si photonic integrated circuits

† Includes both cosmic ray and particle radiations absorbed by silicon-based dosimeter

Those reported ground tests are significantly different from the radiation background on LEO: (1) heavily overdosed compared to LEO space environment. (2) focusing on a single energy level γ or X-ray, while the space cosmic rays cover a broad range of spectra. A similar case is applied to X-ray. (3) Critical particle radiation is much less studied, limited to a few studies of neutron exposure or on passive device studies with negative results. (4) a broad range of energetic protons exposure on actives is missing, (5) limited to DC optoelectronic characterizations (Fig. S6a).

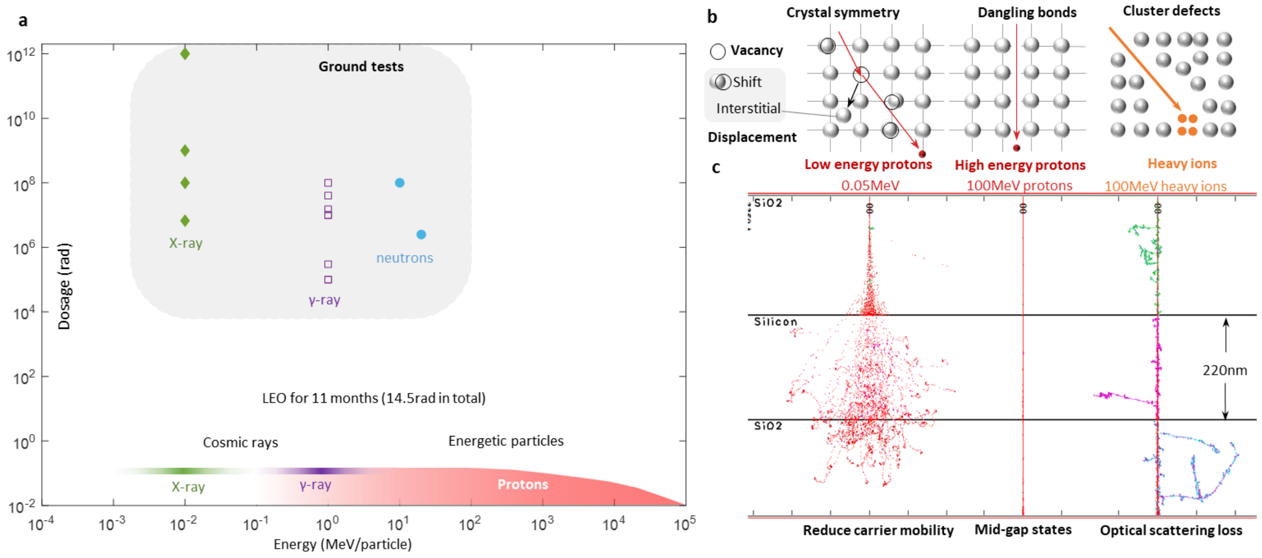

Fig. S6 | Comparison between ground tests and year-long radiation on LEO. (a) Radiation environment comparison between ground tests reported so far and the real space environment (Table S-III). (b) Atomistic pictures of the particle radiation-induced defects in silicon crystalline lattice, for low energy/speed protons produced vacancy and displacement (left), high energy protons induced dangling bonds (middle) and heavy ions resulted in cluster defects (right). (c) Simulated particle trajectory in silicon-on-insulator substrate (220 nm silicon layer), for low-energy protons (left), high-energy protons (middle), and high-energy heavy ions (right). The left one is more available on ground test, while high energy protons and heavy ions are more prevalent in space.

The atomic origin of the particle radiation damage is associated with the creation of defects in single-crystalline lattices, which can be in the form of a change in the crystalline symmetry, formation of silicon vacancies, and interstitial atoms. For low-energy proton exposure, more vacancies and interstitial atoms are created near the device layer, creating carrier scatter centers, and

reducing carrier mobility. After cosmic radiation exposure with low density and high energy proton, the resistance and thus carrier mobility is not changed, but we notice the creation of mid-gap states as carrier recombination centers (increase ideality factor and reduce free carrier lifetime). The cluster defects are more likely to be induced by heavy ions/nuclei, which is the only mechanism that destroys nanostructures and disrupts the waveguide propagation (Fig. S6b-c). It is noted that due to the nanoscale device statistics, we cross-compare multiple characterization results/multiple devices to identify the nanostructured material response (summarized in Table S-IV).

Table S-IV | Radiation induced defect types and silicon photonic device response

	Ionizing radiation (X, γ-rays)		Particle radiation (Protons, α, neutrons)		Heavy ions
Atomic origin	Ground: Mrad	**LEO:** 15 rad	Ground: low energy, high dose	**LEO:** High energy charged particles	**Space only**
Surface oxidation	$\Delta n_{eff}\downarrow$ (2,3)	--	--	--	--
Crystal asymmetry (1)	--	--	$\Delta n_{eff}\uparrow$, Scattering loss in WG (2,3)	$\Delta n_{eff}\uparrow$ Doping dependent $\Delta k\uparrow$ (2-4)	--
Mid-gap states	Reduced EO tuning (5)	--	--	Reduce carrier lifetime (6,7,8)	--
Custer defects	--	--	--	--	High loss (9)

* Observations: (1) Micro-spectroscopy, (2) propagation loss (or Q), (3) Δn_{eff}, (4) doping dependent radiation damage, (5) EO tuning, (6) high speed EO, (7) diode response, (8) optical nonlinearity, (9) MZM with long arm of doped waveguides.

Here we just focus on silicon photonics (single crystalline silicon on insulator substrate). Amorphous materials (such as SiNx, a-Si) are not sensitive to radiation damage [even with upto 10^7 rad γ ray (58), or 10^{15} cm^{-2} α particles (60)] given their highly defective nature. Micro-Raman spectroscopy in section S2 shows broadened bandwidth of the silicon waveguide (device layer embedded under oxide with metal interconnects), suggesting that the crystalline symmetry degradation in some passive waveguide, which is likely to be caused by the high energy charged particles (protons) given such low accumulative dosage (Table S-IV).

REFERENCES

1. R. S. Lu, K. Asada, T. P. Krichbaum, J. Park, F. Tazaki, H. Y. Pu, M. Nakamura, A. Lobanov, K. Hada, K. Akiyama, J. Y. Kim, A ring-like accretion structure in M87 connecting its black hole and jet. *Nature* **616**, 686 (2023).

2. R. E. Lafon, E. Robert, Y. Bai, A. Caroglanian, J. Dailey, N. Desch, H. Garon, S. Hall, R. Miller, D. Paulson, H. Safavi, P. Sekulic, J. V. Speer, P. Thompson, V. C. Wu, Current status of NASA's low-cost optical terminal (LCOT) at Goddard space flight center. *Proc. SPIE* **12413**, 238 (2023).

3. F. I. Khatri, M. Bay, J. King, J. Chang, T. Hudson, R. T. Schulein, O. Mikulina, J. J. Zinchuk, R. J. McGraw, J. Gregory, P. W. Gramm, Optical communications operations concept for the Artemis II crewed mission to the Moon. *SPIE* **12413**, 77 (2023).

4. M. Asghari, A. V. Krishnamoorthy. Silicon photonics: Energy efficient communication. *Nat. Photonics* **5**, 268–270 (2011).

5. P. J. Winzer, D. T. Neilson, From scaling disparities to integrated parallelism: A decathlon for a decade. *J. Light. Technol.* **35**, 1099–1115 (2017).

6. P. Dong, J. Chen, A. Melikyan, T. Fan, T. Fryett, C. Li, J. Chen, and C. Koeppen, "Silicon Photonics for 800G and Beyond," paper presented at the Optical Fiber Communication Conference (OFC) 2022, San Diego, CA, USA, 6–10 March 2022.

7. A. Rizzo, S. Daudlin, A. Novick, A. James, V. Gopal, V. Murthy, Q. Cheng, B. Y. Kim, X. Ji, Y. Okawachi, M. van Niekerk, Petabit-scale silicon photonic interconnects with integrated Kerr frequency combs. *IEEE J. Sel. Top. Quantum Electron.* **29**, 3700120 (2023).

8. C. Rogers, A. Y. Piggott, D. J. Thomson, R. F. Wiser, I. E. Opris, S. A. Fortune, A. J. Compston, A. Gondarenko, F. Meng, X. Chen, G. T. Reed, A universal 3D imaging sensor on a silicon photonics platform. *Nature* **590**, 256–261 (2021).

9. A. H. Atabaki, S. Moazeni, F. Pavanello, H. Gevorgyan, J. Notaros, L. Alloatti, W. T. Wade, C. Sun, S. A. Kruger, H. Meng, K. A. Qubaisi, I. Wang, B. Zhang, A. Khilo, C. V. Baiocco, M. A. Popović, V. M. Stojanović, R. J. Ram, Integrating photonics with silicon nanoelectronics for the next generation of systems on a chip. *Nature* **556**, 349–354 (2018).

10. M. Krainak, M. Stephen, E. Troupaki, S. Tedder, B. Reyna, J. Klamkin, H. Zhao, B. Song, J. Fridlander, N. Tran, J. E. Bowers, K. Bergman, M. Lipson, A. Rizzo, I. Datta, N. Abrams, S. Mookherjea, S. T. Ho, Q. Bei, Y. Huang, Y. Tu, B. Moslehi, J. Harris, A. Matsko, A. Savchenkov, G. Liu, R. Proietti, S. J. B. Yoo, L. Johansson, C. Dorrer, F. R. Arteaga-Sierra, J. Qiao, S. Gong, T. Gu, O. J. Ohanian, X. Ni, Y. Ding, Y. Duan, H. Dalir, R. T. Chen, V. J. Sorger, T. Komljenovic. Integrated photonics for NASA applications. *Proc. SPIE.* **10899**, 10.1117/12.2509808 (2019).

11. G. Brunetti, N. Saha, G. Campiti, A. Toma, N. Sasanelli, F. Hassan, M. N. Armenise, C. Ciminelli, Integrated photonics for NewSpace. *ApplePies* **1036**, 294–299 (2022).

12. G. N. Tzintzarov, S. G. Rao, J. D. Cressler, Integrated silicon photonics for enabling next-generation space systems. *Photonics* **8**, 131 (2021).

13. T. J. Logue, J. Pelton, "Overview of commercial small satellite systems in the "New Space" age" in *Handbook of Small Satellites: Technology, Design, Manufacture, Applications, Economics and Regulation*, J. N. Pelton, S. Madry, Eds. (Springer Press, 2020).

14. F. K. Reed, N. Ezell, M. N. Ericson, C. L. Britton Jr, "Radiation hardened electronics for reactor environments" (Tech. Rep. ORNL/TM-2020/1776, Oak Ridge National Laboratory, 2020).

15. S. Girard, A. Morana, A. Ladaci, T. Robin, L. Mescia, J.-J. Bonnefois, M. Boutillier, J. Mekki, A. Paveau, B. Cadier, E. Marin, Recent advances in radiation-hardened fiber-based technologies for space applications. *J. Opt.* **20**, 093001 (2018).

16. J.L. Barth, *Space and Atmospheric Environments: From Low Earth Orbits to Deep Space*, in *Protection of Materials and Structures from Space Environment*, J. Kleiman, M. Takagawa, Y. Kimoto, Eds. (Springer, 2004), vol. 5.

17. J. W. Henke, A. S. Raja, A. Feist, G. Huang, G. Arend, Y. Yang, F. J. Kappert, R. N. Wang, M. Möller, J. Pan, J. Liu, O. Kfir, C. Ropers, T. J. Kippenberg Integrated photonics enables continuous-beam electron phase modulation. *Nature* **600**, 653–658 (2021).

18. A. Ben Hayun, O. Reinhardt, J. Nemirovsky, A. Karnieli, N. Rivera, I. Kaminer. Shaping quantum photonic states using free electrons. *Sci. Adv.* **7**, eabe4270 (2021).

19. S. Bhandaru, S. Hu, D. M. Fleetwood, S. M. Weiss, Total ionizing dose effects on silicon ring resonators. *IEEE Trans. Nucl. Sci.* **62**, 323–328 (2015).

20. N. Boynton, M. Gehl, C. Dallo, A. Pomerene, A. Starbuck, D. Hood, P. Dodd, S. Swanson, D. Trotter, C. DeRose, and A. Lentine, Gamma radiation effects on passive silicon photonic waveguides using phase sensitive methods. *Opt. Express* **28**, 35192–35201 (2020).

21. G. B. Hoffman, M. Gehl, N. J. Martinez, D. C. Trotter, A. L. Starbuck, A. Pomerene, C. M. Dallo, D. Hood, P. E. Dodd, S. E. Swanson, C. M. Long, The effect of gamma radiation exposure on active silicon photonic device performance metrics. *IEEE Trans. Nucl. Sci.* **66**, 801–809 (2019).

22. Z. Ahmed, L. T. Cumberland, N. N. Klimov, I. M. Pazos, R. E. Tosh, R. Fitzgerald, Assessing radiation hardness of silicon photonic sensors. *Sci. Rep.* **8**, 13007 (2018)

23. G. N. Tzintzarov, J. W Teng, D. Nergui, B. L Ringel, S. D. Lalumondiere, D. M Monahan, A. Little, J. D Cressler, Direct measurement of total-ionizing-dose-induced phase shifts in commercially available, integrated silicon-photonic waveguides. *IEEE Trans. Nucl. Sci.* **70**, 2116–2124(2023)

24. M. Zeiler, S. S. E. Nasr-Storey, S. Detraz, A. Kraxner, L. Olantera, C. Scarcella, C. Sigaud, C. Soos, J. Troska, F. Vasey, Radiation damage in silicon photonic Mach-Zehnder modulators and photodiodes. *IEEE Trans. Nucl. Sci.* **64**, 2794–2801 (2017)

25. S. S. Nasr-Storey, F. Boeuf, C. Baudot, S. Detraz, J. M. Fedeli, D. Marris-Morini, L. Olantera, G. Pezzullo, C. Sigaud, C. Soos, and J. Troska, F. Vasey, L. Vivien, M. Zeiler, M. Ziebell, Effect of radiation on a Mach-Zehnder interferometer silicon modulator for HL-LHC data transmission applications. *IEEE Trans. Nucl. Sci.* **62**, 329–335 (2015).

26. M. Lalovic, C. Scarcella, A. Bulling, S. Detraz, L. Marcon, L. Olanterä, T. Prousalidi , U. Sandven, C. Sigaud, C. Soos, J. Troska, Ionizing radiation effects in silicon photonics modulators. *IEEE Trans. Nucl. Sci.* **69**, 1521–1526 (2022).

27. J. R. Cummings, R. A. Mewaldt; R. S. Selesnick; E. C. Stone; T. T. von Rosenvinge, New evidence for anomalous cosmic rays trapped in the magnetosphere, *Proceedings of the 23rd International Cosmic Ray Conference*, (ICRC23, 1993).

28. O. Adriani, G. C. Barbarino, G. A. Bazilevskaya, R. Bellotti, M. Boezio, E. A. Bogomolov, M. Bongi, V. Bonvicini, S. Bottai, A. Bruno, F. Cafagna, D. Campana, R. Carbone, P. Carlson, M. Casolino, G. Castellini, C. De Donato, C. De Santis, N. De Simone, V. Di Felice, V. Formato, A. M. Galper, A. V. Karelin, S. V. Koldashov, S. Koldobskiy, S. Y. Krutkov, A. N. Kvashnin, A. Leonov, V. Malakhov, L. Marcelli, M. Martucci, A. G. Mayorov, W. Menn, M. Mergé, V. V. Mikhailov, E. Mocchiutti, A. Monaco, N. Mori, R. Munini, G. Osteria, F. Palma, B. Panico, P. Papini, M. Pearce, P. Picozza, M. Ricci, S. B. Ricciarini, R. Sarkar, V. Scotti, M. Simon, R. Sparvoli, P. Spillantini, Y. I. Stozhkov, A. Vacchi, E. Vannuccini, G. I. Vasilyev, S. A. Voronov, Y. T. Yurkin, G. Zampa, N. Zampa, V. G. Zverev. Trapped proton fluxes at low Earth orbits measured by the PAMELA experiment. *ApJL* **799**, L4 (2015).

29. D. Nikolić, A. Vasić, I. Fetahović, K. Stanković, P. Osmokrović, in *Photodiode Behavior in Radiation Environment* (State Univ. of Novi Pazar, 2011) vol. 3, pp. 27–34.

30. V. Brasch, Q. F. Chen, S. Schiller, T. J. Kippenberg, Radiation hardness of high-Q silicon nitride microresonators for space compatible integrated optics. *Opt. Express* **22**, 30786–30794 (2014).

31. G. Davies, S. Hayama, L. Murin, R. Krause-Rehberg, V. Bondarenko, A. Sengupta, C. Davia, A. Karpenko, Radiation damage in silicon exposed to high-energy protons. *Phys. Rev. B* **73**, 165202 (2006).

32. T. Vogl, K. Sripathy, A. Sharma, P. Reddy, J. Sullivan, J. R. Machacek, L. Zhang, F. Karouta, B. C. Buchler, M. W. Doherty, Y. Lu, P. K. Lam, Radiation tolerance of two-dimensional material-based devices for space applications. *Nat. Commun.* **10**, 1202 (2019).

33. S. Muraro, G. Battistoni, A. C. Kraan, Challenges in Monte Carlo simulations as clinical and research tool in particle therapy: A review. *Front. Phys.* **8**, 567800 (2020).

34. A. Panglosse, P. Martin-Gonthier, O. Marcelot, C. Virmontois, O. Saint-Pé, P. Magnan, Dark count rate modeling in single-photon avalanche diodes. *IEEE TCAS-I* **67**, 1507–1515 (2020).

35. J. Wen, W. J. Wang, X. R. Chen, N. Li, X. S. Chen, W. Lu, Origin of large dark current increase in InGaAs/InP avalanche photodiode. *J. Appl. Phys.* **123**, 161530 (2018).

36. S. Girard, A. Alessi, N. Richard, L. Martin-Samos, V. De Michele, L. Giacomazzi, S. Agnello, D. Di Francesca, A. Morana, B. Winkler, I. Reghioua, Overview of radiation-induced point defects in silica-based optical fibers. *Rev. Phys.* **4**, 100032 (2019).

37. F. De Leonardis, B. Troia, C. E. Campanella, F. Prudenzano, V. M. Passaro, Modeling of radiation effects in silicon photonic devices. *IEEE Trans. Nucl. Sci.* **62**, 2155–2168 (2015).

38. H. Bethe, W. Heitler, On the stopping of fast particles and on the creation of positive electrons. *Proc. R. Soc. Lond. A.* **146**, 83–112 (1934).

39. P. Dong, J. H. Sinsky, C. Gui, Coplanar-waveguide-based silicon Mach-Zehnder modulator using a meandering optical waveguide and alternating-side PN junction loading. *Opt. Lett.* **41**, 4401 (2016), 4404.

40. S. M. Sze, K. K. Ng, *Physics of Semiconductor Devices* (John Wiley & Sons, 2006).

41. P. E. Barclay, K. Srinivasan, O. Painter, Nonlinear response of silicon photonic crystal micro-resonators excited via an integrated waveguide and fiber taper. *Opt. Express* **13**, 801–820 (2005).

42. T. Gu, N. Petrone, J. F. McMillan, A. van der Zande, M. Yu, G. Q. Lo, D. L. Kwong, J. Hone, C. W. Wong, Regenerative oscillation and four-wave mixing in graphene optoelectronics. *Nat. Photonics* **6**, 554–559 (2012).

43. G. Messenger, A summary review of displacement damage from high energy radiation in silicon semiconductors and semiconductor devices. *IEEE Trans. Nucl. Sci.* **39**, 468–473 (1992).

44. V. Eremin, Z. Li, Carrier drift mobility study in neutron irradiated high purity silicon. *Nucl. Instrum. Methods Phys. Res., Sect. A* **362**, 338–343 (1995).

45. N. Stoffle, L. Pinsky, M. Kroupa, S. Hoang, J. Idarraga, C. Amberboy, R. Rios, J. Hauss, J. Keller, A. Bahadori, E. Semones, D. Turecek, J. Jakubek, Z. Vykydal, S. Pospisil, Timepix-based radiation environment monitor measurements aboard the International Space Station. *Nucl. Instrum. Methods Phys. Res., Sect. A* **782**, 143–148 (2015).

46. A. J. Mercante, S. Shi, P. Yao, L. Xie, R. M. Weikle, D. W. Prather. Thin film lithium niobate electro-optic modulator with terahertz operating bandwidth, *Opt. Express* **26**, 14810–14816 (2018).

47. Y. Shi, L. Yan, A. E. Willner, High-speed electrooptic modulator characterization using optical spectrum analysis. *J. Light. Technol.* **21**, 2358–2367 (2003).

48. B. E. Little, S. T. Chu, H. A. Haus, J. A. F. J. Foresi, J.-P. Laine, Microring resonator channel dropping filters. *J. Light. Technol.* **15**, 998–1005 (1997).

49. Y. Akahane, T. Asano, B.-S. Song, S. Noda, Fine-tuned high-Q photonic-crystal nanocavity. *Opt. Express* **13**, 1202–1214 (2005).

50. A. Griffith, J. Cardenas, B. Poitras, M. Lipson, High quality factor and high confinement silicon resonators using etchless process. *Opt. Express* **20**, 21341–21345 (2012)

51. P. Rabiei, W. Steier, L. Dalton, Polymer micro-ring filters and modulators. *J. Light. Technol.* **20**, 1968–1975 (2002).

52. S. Nishibe, T. Sasaki, H. Harima, K. Kisoda, T. Yamazaki, W. S. Yoo, Raman study on the process of Si advanced integrated circuits, in the *14th IEEE International Conference on Advanced Thermal Processing of Semiconductors* (IEEE, 2006), pp. 211–215.

53. T. Gu, M. A. El-Emawy, K. Yang, A. Stintz, L. F. Lester, Resistance to edge recombination in GaAs-based dots-in-a-well solar cells. *Appl. Phys. Lett.* **95**, 261106 (2009).

54. D. Mao, C. Cheng, F. Wang, Y. Xiao, T. Li, L. Chang, A. Soman, T. Kanenen, X. Zhang, M. Krainak, P. Dong, T. Gu, Device architectures for low voltage and ultrafast graphene integrated phase modulators. *IEEE J Sel. Top. Quantum Electron.* **27**, 3400309 (2021).

55. G. Brunetti, I. McKenzie, F. Dell'Olio, M. N. Armenise, C. Ciminelli, Measured radiation effects on InGaAsP/InP ring resonators for space applications, *Opt. Express* **27**, 24434–24444 (2019).

56. S. Grillanda, V. Singh, V. Raghunathan, F. Morichetti, A. Melloni, L. Kimerling, A. M. Agarwal, Gamma radiation effects on silicon photonic waveguides. *Opt. Lett.* **41**,3053–3056 (2016).

57. Q. Du, Y. Huang, O. Ogbuu, W. Zhang, J. Li, V. Singh, A. M. Agarwal, J. Hu, Gamma radiation effects in amorphous silicon and silicon nitride photonic devices. *Opt. Lett.* **42**, 587–590 (2017).

58. Q. Du, J. Michon, B. Li, D. Kita, D. Ma, H. Zuo, S. Yu, T. Gu, A. Agarwal, M. Li, J. Hu, Real-time, in situ probing of gamma radiation damage with packaged integrated photonic chips. *Photon. Res.* **8**, 186–193 (2020).

59. Y. Zhou, D. Bi, S. Wang, L. Wu, Y. Huang, E. Zhang, D. M. Fleetwood, A. Wu, High energy irradiation effects on silicon photonic passive devices. *Opt. Express* **30**, 4017–4027 (2022).

60. F. Morichetti, S. Grillanda, S. Manandhar, V. Shutthanandan, L. Kimerling, A. Melloni, A. M. Agarwal, Alpha radiation effects on silicon oxynitride waveguides, *ACS Photonics* **3**, 1569–1574 (2016).
